# Distinct tonic inhibition and its plasticity in hippocampal OLM and non-OLM somatostatin-positive interneurons

**DOI:** 10.3389/fnmol.2026.1934043

**Published:** 2026-10-30

**Authors:** Patrycja Droździel, Katarzyna Lebida, Jerzy W. Mozrzymas

**Affiliations:** Department of Biophysics and Neuroscience, Wroclaw Medical University, Wroclaw, Poland

**Keywords:** tonic inhibition, GABA_A_ receptors, interneurons, hippocampus, inhibitory plasticity

## Abstract

Extrasynaptic GABA_A_ receptors (GABA_A_Rs) mediating tonic inhibition are critically involved in the regulation of neuronal activity, synaptic plasticity and hippocampus-dependent learning and memory. Tonic inhibition itself was found to undergo plastic changes but the description of this plasticity in distinct subclasses of hippocampal interneurons (INs) remains incomplete. In the present study, we combined whole-cell electrophysiology with morphological reconstruction of biocytin-filled neurons to investigate plasticity of tonic GABAergic currents, induced by brief (3 min) NMDA administration, in oriens-lacunosum moleculare (OLM) and non-OLM subclasses of somatostatin-positive (SST+) INs in the hippocampal CA1 region. We found that, under control conditions, tonic current density was significantly larger in OLM than in non-OLM INs. Brief NMDA receptor activation significantly enhanced tonic current density in a subset of non-OLM SST+ INs, distinguished as the upper-median in the cumulative distribution, whereas tonic current density in OLM cells showed no plastic changes upon NMDA treatment. Since in the hippocampus, GABAergic plasticity often involves α5-containing GABA_A_Rs (α5-GABA_A_Rs), we used the selective α5-GABA_A_R inverse agonist L-655,708 and found that NMDA treatment increased the relative contribution of α5-GABA_A_Rs to tonic currents in non-OLM INs. These data altogether demonstrate that, whereas non-OLM SST+ INs show lower tonic current density than OLM cells, the former subgroup is characterized by greater susceptibility to NMDA-induced changes in tonic current density and these plastic changes are associated with an increased contribution of α5-GABA_A_Rs. The presented observations suggest a clear functional specialization of non-OLM and OLM SST+ INs in the regulation of hippocampal network activity.

## 1 Introduction

The hippocampus plays a pivotal role in learning, memory formation, and spatial navigation (Clark et al., 2000; Ekstrom and Hill, 2023; Madhav et al., 2024; Kwon et al., 2025), and proper hippocampal function relies on a precise balance between excitatory and inhibitory synaptic activity. While much attention has traditionally focused on fast, phasic synaptic inhibition mediated by transient γ-aminobutyric acid (GABA) release, growing evidence highlights the importance of tonic inhibitory currents, a persistent form of inhibition that activates high-affinity extrasynaptic receptors by ambient low GABA concentrations (Farrant and Nusser, 2005). The heterogeneity of GABA_A_R subunits and their diverse assembly configurations give rise to multiple receptor subtypes with distinct properties, contributing to diversified GABAergic signaling, both phasic and tonic, across brain regions and cell types. In the hippocampus, tonic inhibition is predominantly mediated by receptors containing α5 and δ subunits (Caraiscos et al., 2004; Glykys et al., 2008). These receptors generate a persistent chloride conductance that can modulate neuronal input resistance (Ri), membrane time constants (τm), and the gain of neuronal input–output functions, thereby shaping dendritic integration, action potential firing, and overall information processing (Farrant and Nusser, 2005; Lee and Maguire, 2014; Jacob, 2019). Furthermore, accumulating evidence indicates that tonic inhibition and α5 subunit-containing GABA_A_Rs (α5-GABA_A_Rs) are crucial for synaptic plasticity as well as hippocampus-dependent learning and memory (Collinson et al., 2002; Martin et al., 2009). Recent studies have demonstrated that tonic inhibition is not a static background process but displays substantial plasticity, enabling inhibitory tone to adapt to neuronal activity, development, or pathological states such as seizure-like activity (Scimemi et al., 2005; Qi et al., 2006; Groen et al., 2014). Importantly, alterations of tonic inhibition were found to affect excitatory synaptic plasticity in hippocampal circuits, thereby positioning this type of inhibition as a potential metaplastic controller of learning rules (Martin et al., 2010; Dembitskaya et al., 2020). Several other studies have further implicated α5-GABA_A_Rs in the regulation of glutamatergic plasticity (Cheng et al., 2006; Salesse et al., 2011; Davenport et al., 2021) and in synapse- and cell type-dependent inhibitory synaptic plasticity (Wiera et al., 2021; Brzdąk et al., 2023). Moreover, inhibitory long-term potentiation (iLTP) induced by N-methyl-D-aspartic acid (NMDA) receptor activation in somatostatin-expressing interneurons (SST+ INs) is accompanied by prolonged miniature inhibitory postsynaptic current (mIPSC) decay due to synaptic incorporation of α5-GABA_A_Rs (Brzdąk et al., 2023). Interestingly, NMDA-driven heterosynaptic inhibitory plasticity also triggers alterations in tonic inhibition across CA1 pyramidal cells (PCs) and INs, including SST+ INs (Wyroślak et al., 2021, 2023). Specifically, while parvalbumin-positive (PV+) cells exhibit a reduction in tonic currents following plasticity induction, SST+ INs display an enhancement of tonic currents mediated by an increased content of α5-GABA_A_Rs (Wyroślak et al., 2023).

SST+ INs constitute a heterogeneous population (Pelkey et al., 2017; Honoré et al., 2021) and in the CA1 region they preferentially target the dendrites of PCs locally, but their axonal arbors exhibit layer-specific preferences aligned with distinct glutamatergic input pathways (Klausberger and Somogyi, 2008; Udakis et al., 2020). Among CA1 SST+ INs, oriens-lacunosum moleculare (OLM) cells constitute the most abundant subgroup (Asgarian et al., 2019). These neurons exhibit a unique morphology: they receive feedback excitation from CA1 in stratum oriens (SO) and provide targeted inhibition specifically to the distal dendrites of CA1 PCs in the stratum lacunosum-moleculare (SLM). Concurrently, they inhibit INs targeting PCs disinhibiting the proximal dendrites of the principal neurons. Thus, OLM cells are able to modulate excitatory inputs from both the CA3 region and entorhinal cortex onto CA1 PCs (Leão et al., 2012). OLM INs have emerged as key regulators of information flow within hippocampal circuitry, playing essential roles in spatial memory, contextual fear memory, and object recognition (Siwani et al., 2018; Honoré and Lacaille, 2022; Udakis et al., 2025). The heterogeneity of SST+ INs is probably crucial for the modulation of hippocampal network activity, particularly with regard to LTP and memory formation (Lovett-Barron et al., 2014; Siwani et al., 2018; Honoré et al., 2021). This diversity also implies that plastic changes may not be uniform across all SST+ IN subtypes. However, it remains unknown whether distinct SST+ IN populations differ in the plasticity of tonic inhibitory currents. Therefore, understanding how NMDA-induced plasticity differentially modulates tonic current density in specific subgroups of SST+ INs is essential for elucidating their precise contributions to learning and memory. Addressing this question may provide insight into how individual interneuron subpopulations regulate the flow of information within the hippocampal network and could shed light on whether their dysfunction contributes to pathological disorders.

We thus used the protocol of transient (3 min) NMDA application, known to induce heterosynaptic iLTP, to study plasticity of tonic inhibition in two subtypes of hippocampal SST+ INs: OLM and non-OLM cells, using combined electrophysiological and immunofluorescence techniques. We found clear differences in tonic current density between these two SST+ IN subtypes in control conditions, underscoring their functional heterogeneity. Notably, brief NMDA receptor activation triggered cell type-specific plasticity changes in tonic inhibition: tonic current density remained unchanged in OLM cells but increased significantly in a subset of non-OLM cells (upper-median group). Furthermore, the NMDA-induced alteration in tonic current density was associated with a significant increase in the percentage contribution of α5-GABA_A_Rs in the total tonic current density.

Together, these findings indicate that brief NMDA receptor activation induces cell type-specific plasticity of tonic inhibition in a subset of SST+ INs and is accompanied by an increased relative contribution of α5-GABA_A_Rs, highlighting potential differences in the regulation of tonic inhibition among SST+ IN subtypes.

## 2 Materials and methods

### 2.1 Animals

Animals were housed under a 12/12 h light/dark cycle with water and food supplied ad libitum at the animal facility of the Wroclaw Medical University. For the experiments, SST-Cre (JAX 013044, STOCK Ssttm2.1(cre)Zjh/J; RRID: IMSR_JAX:013044) x Ai14 (JAX 007914, B6. Cg-Gt(ROSA)26Sortm14(CAG-tdTomato)Hze/J; RRID: IMSR_JAX:007914) mice of both sexes, aged 20–24 days, were used. The use of genetically modified animals was authorized by the Polish Ministry of the Environment (approval no. 69/2023). All procedures were conducted in accordance with the Polish Act on the Protection of Animals Used for Scientific or Educational Purposes (Journal of Laws 2015, item 266) and with the EU Directive 2010/63/EU. Experiments were performed in accordance with the ARRIVE guidelines.

### 2.2 Slice preparation

Mice were anesthetized with 4% isoflurane delivered using a TAIJI-IE small animal anesthesia system (RWD Life Science, Shenzhen, China) with oxygen (0.5–1.0 L/min) provided by a ROC-5A veterinary oxygen concentrator (RWD Life Science, Shenzhen, China). After decapitation, the brain was removed and placed in a chamber containing chilled (approximately 0 °C) artificial cerebrospinal fluid (ACSF), composed of (in mM): 119 NaCl, 26.3 NaHCO_3_, 11 glucose, 2.5 KCl, 1 NaH_2_PO_4_, 1.3 MgSO_4_, and 2.5 CaCl_2_ (295 mOsm, pH 7.4). The buffer was continuously bubbled with carbogen (95% O_2_+ 5% CO_2_). Next, the brain was sectioned into 350 μm slices using a vibrating microtome (Leica VT1200S, Germany). Slices were transferred to a beaker containing the same ACSF solution as described above and incubated for 1 h at room temperature. During relaxation, the solution was bubbled with carbogen (95% O_2_, 5% CO_2_) to maintain proper oxygenation (Wyroślak et al., 2023).

### 2.3 Electrophysiological recordings

After incubation, a single slice was placed in a recording chamber and continuously perfused with the ACSF at a flow rate of 2.0–3.0 mL/min. Whole-cell patch-clamp recordings were performed at room temperature from SST+ INs located in the SO of the CA1 region of the hippocampus. These cells were identified by detecting tdTomato fluorescence using a Nikon ECLIPSE FN1 upright fluorescence microscope (Nikon Corporation, Tokyo, Japan) equipped with CFI Plan 4 × (NA 0.10) and CFI Apochromat NIR 40 × water-immersion (NA 0.80) objectives. The 4 × objective was used for initial slice navigation, whereas the 40 × water-immersion objective was used for cell identification and whole-cell patch-clamp recordings. For experiments, borosilicate glass pipettes with a resistance of 3–4 MΩ were filled with an intracellular solution containing (in mM): 10 potassium gluconate, 125 KCl, 1 EGTA, 10 HEPES, 4 MgATP, 5 sucrose, adjusted to pH 7.25 and 295 mOsm (Marsden et al., 2007). The same KCl-based internal solution was used throughout the whole-cell recording, including the initial current-clamp characterization and subsequent voltage-clamp measurements of tonic GABA_A_R-mediated currents. The high intracellular Cl^−^ concentration increases the Cl^−^ driving force, thereby facilitating the detection and quantification of GABA_A_R-mediated currents. Using the same internal solution allowed both measurements to be obtained from the same neuron within a single whole-cell recording. Biocytin (0.3%) was added to the intracellular solution to enable subsequent visualization of the recorded cells. Recordings were obtained at a holding potential of −70 mV using a MultiClamp 700B amplifier and a Digidata 1550B digitizer (Molecular Devices). Each experiment began with an assessment of the firing properties of the recorded neuron in current clamp mode using a series of 300 ms current injections ranging from −200 pA to +200 pA (Figures 1C,D). Tetrodotoxin (TTX, 1 μM) and 6,7-dinitroquinoxaline-2,3-dione (DNQX, 20 μM) were added to external solution to block voltage-gated sodium channel-dependent action potentials and fast glutamatergic synaptic currents mediated by AMPA/kainate receptors, respectively (Table 1; Marchionni et al., 2009). A 10 min recording period was then used to establish the baseline. Following baseline recording, one of four experimental protocols was implemented. In the first group, etomidate (3 μM) was applied to potentiate tonic inhibitory currents, followed by the non-specific GABA_A_R antagonist picrotoxin (PTX, 100 μM) to block GABAergic currents. In the second protocol, slices were exposed to NMDA (20 μM) for 3 min prior to sequential application of etomidate and PTX to induce synaptic iLTP (Marsden et al., 2007). In the third group, the selective inverse agonist of α5-GABA_A_R L-655,708 (20 nM), was applied after etomidate administration but before PTX. In the fourth protocol, NMDA, etomidate, L-655,708, and PTX were applied sequentially. Each protocol was performed in two distinct neuronal populations: OLM and non-OLM INs, identified according to their electrophysiological and morphological characteristics. Groups 1 and 3 served as the respective control conditions.

**Figure 1:**
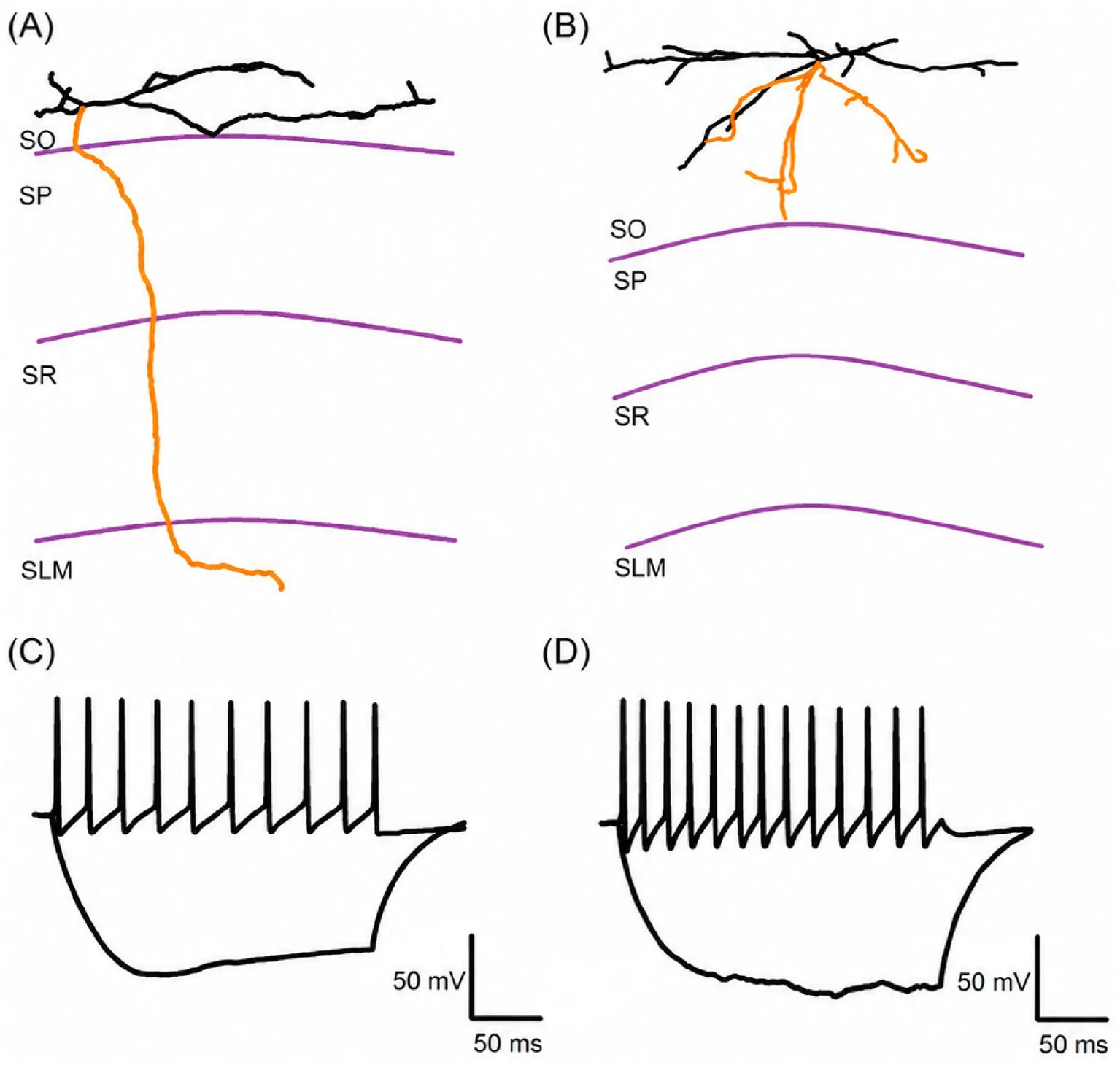
Reconstructions of fluorescently labeled SST+ INs and representative firing patterns. **(A)** and **(B)** Morphological reconstructions of the OLM and non-OLM INs, respectively. Neurons were filled with biocytin and images were generated using neuTube. Axons are marked in orange and dendrites in black. **(C)** and **(D)** Representative firing patterns of cells included in the OLM and non-OLM groups, respectively. The upper and lower traces represent the cell’s response to a depolarizing and hyperpolarizing stimulus, respectively. Note that in OLM cells, a significantly larger sag potential than that in non-OLM INs was observed. SO, stratum oriens; SP, stratum pyramidale; SR, stratum radiatum; SLM, stratum lacunosum-moleculare.

**Table 1:** Sources and catalogue numbers of used reagents.

Reagent	Source	Identifier
Tetrodotoxin	Latoxan	Cat# L8503
6,7-dinitroquinoxaline-2,3-dione	Tocris Bioscience	Cat# 2312
N-methyl-D-aspartic acid	Sigma-Aldrich	Cat# M3262
Etomidate	Sigma	Cat# E6530
L-655,708	Tocris Bioscience	Cat# 1327
Picrotoxin	Sigma	Cat# P1675
Normal horse serum	Thermo Fisher Scientific	Cat# 16050130
Biocytin	Tocris Bioscience	Cat# 3349
Streptavidin Alexa Fluor 488 conjugate	Invitrogen	AB_2315383

### 2.4 Immunofluorescence staining

After the electrophysiological recordings, slices were transferred to a fixative solution containing 4% paraformaldehyde, 0.2% picric acid, and phosphate-buffered saline (PBS) and fixed at 4 °C for 24 h. Following fixation, slices were washed three times in PBS for 15 min each. The staining procedure began with a 15 min incubation in PBS containing 0.5% Triton X-100 to permeabilize the tissue. Slices were then incubated for 1 h at room temperature on a shaker in a blocking solution composed of 5% normal horse serum (NHS) and 0.5% Triton X-100 in PBS to reduce nonspecific binding. Subsequently, slices were incubated with streptavidin-conjugated Alexa Fluor 488 diluted in PBS containing 0.5% Triton X-100 and 3% NHS. This incubation was performed for 24 h at 4 °C on a shaker. After incubation, slices were rinsed three times with PBS containing 0.5% Triton X-100 and mounted onto glass slides using Fluoroshield mounting medium. Cells were visualized using confocal microscopy and their morphological features were reconstructed using neuTube software (Jabłońska et al., 2025).

### 2.5 Cell visualization and reconstruction

Visualization of cells was performed using an Olympus Fluoview 3,000 laser-scanning confocal microscope at Wroclaw Medical University, Poland. Images of 1 μm optical sections were captured as Z-stacks using a 10 × objective and a 488 nm excitation laser. Neuronal reconstruction was carried out using neuTube software. The 3D visualization module was used to examine neuronal structures in detail, including the soma, axon, and dendritic branches. Cell morphology was reconstructed using semimanual tracing (Ascoli et al., 2007; Feng et al., 2015).

### 2.6 Data analysis

Efforts were made to maintain comparable sample sizes across all experimental and control groups. No formal randomisation was applied for the allocation of experimental units to experimental groups. Animals were assigned based on their genotype and availability. The experimenter was aware of the genotype during animal selection and throughout the experimental procedures. Data analysis was performed according to predefined criteria, and all recordings were analyzed using consistent procedures. Brain slices were collected from both dorsal and ventral hippocampal regions, and no distinction between these subregions was made during subsequent analyses. Tissue samples were obtained from mice of both sexes, and no sex-based segregation was applied in data evaluation. Electrophysiological data were analyzed using Clampfit software (pClamp 10.7, Molecular Devices Corporation, USA). Access resistance was monitored during the recordings. Cells were discarded from further analysis if resistance changed by more than 20%. The following electrophysiological parameters were analyzed: resting membrane potential (RMP, mV), τm (ms), action potential (AP) threshold (APt, mV), AP amplitude (mV), firing frequency (Hz), spike half-width (ms), afterhyperpolarization (AHP) amplitude (mV), sag potential (mV) and Ri (MΩ). The RMP was measured while the neuron was maintained at rest in the absence of injected current (*I* = 0 pA). The τm was estimated by fitting a single-exponential function to the rising phase of the voltage response evoked by a −50 pA hyperpolarizing current injection. The APt was defined as the membrane potential at which dV/dt reached 20 mV/ms, and AP amplitude as the difference between the APt and the peak of the first evoked spike. Firing frequency was calculated as the number of APs generated within a 300-ms window, at the 100 pA current step, normalized to the duration of this interval. Spike half-width was measured at half-maximal amplitude of the first AP (Lebida and Mozrzymas, 2017). AHP amplitude was defined as the difference between the most negative membrane potential following the first spike and the APt. Sag potential was determined from responses to a −200 pA hyperpolarizing current step by calculating the difference between the peak negative voltage deflection and the steady-state membrane potential. Ri was calculated from the change in membrane potential (ΔV) in response to hyperpolarizing current injections (−25 and −50 pA) according to Ohm’s law and expressed as the mean of the obtained values (Song et al., 2015; Wyroślak et al., 2023). Tonic current density was calculated from the shift in holding current observed after PTX application relative to the baseline holding current measured after etomidate application. The tonic current density was calculated from stable 5–6 s recording intervals selected using the same procedure for all recordings. For baseline measurements, slices were perfused with TTX and DNQX for 10 min. In cells subjected to NMDA-induced plasticity, NMDA was applied for 3 min, followed by 18 min of stable recording. For pharmacological manipulation of tonic currents, etomidate was applied for 8 min, L-655,708 for 10 min, and PTX for 15 min (Supplementary Figure S1). Within each cell, recording segments of the same or very similar duration were selected while avoiding mIPSCs and periods of apparent recording noise or instability. Values were normalized to whole-cell membrane capacitance (Cm), as previously described (Urban-Ciecko et al., 2010; Wyroślak et al., 2021). All data are presented as the mean ± SEM. Classification of recorded cells into OLM and non-OLM groups was based on the analysis of firing patterns, with particular attention to sag potential, together with examining cell morphologies reconstructed with neuTube. The complete classification procedure and the parameter values used are provided in the Results section.

### 2.7 Statistical analysis

Statistical analysis was carried out using SigmaPlot (Systat Software, Inc.). N represents the number of biologically independent animals (total N = 46 mice), whereas n represents the number of analyzed cells. The normality of data distribution was assessed using the Shapiro–Wilk test before selecting the appropriate statistical test. For normally distributed data, differences between two independent groups were evaluated using an unpaired Student’s *t*-test, whereas data that did not meet the assumption of normality were analyzed using the Mann–Whitney *U* test. Comparisons between paired datasets (before and after L-655,708 application) were performed using a paired *t*-test for normally distributed data or the Wilcoxon signed-rank test when the assumption of normality was not met. Differences were considered statistically significant when *p* < 0.05. To quantify the tonic current mediated by α5-GABA_A_Rs, the parameter %ΔI (L-655,708) was calculated as:


$$\begin{array}{l} \%\mathrm{\Delta I}\;(L-655,708)\\ =((I_{\text{etomidate}}-I_{L-655,708})/(I_{\text{etomidate}}-I_{\mathrm{PTX}}))*100\% \end{array}$$

where I_etomidate_ − I_L-655,708_ reflects the absolute value of the component of tonic current density sensitive to L-655,708 (mediated by α5-GABA_A_Rs) and I_etomidate_ - I_PTX_ represents the total tonic current density. For statistical analysis of NMDA-induced changes in tonic current density in OLM and non-OLM cells, GraphPad Prism software was used. Frequency distribution plots were generated, and differences between distributions were assessed using the Kolmogorov–Smirnov test. To explore the marked heterogeneity in tonic current density among non-OLM INs, the cells were divided into two groups based on the median value (below and above the median). The median value was calculated separately for the control and NMDA datasets, based on the distribution of tonic current density values. Comparisons between the corresponding control and NMDA-treated subgroups were performed using the Mann–Whitney *U* test. For clarity, the data groups below and above the median are referred to as lower-median and upper-median, respectively.

## 3 Results

### 3.1 Morphological reconstructions and firing pattern analysis identify two subgroups of SST+ INs

SST+ INs represent a heterogeneous population of inhibitory neurons characterized by substantial variations in morphology, synaptic connectivity, and electrical properties. Taking this into account, prior to characterizing the tonic inhibition and its NMDA-induced plasticity in distinct SST+ INs, we carried out morphological reconstructions and firing pattern analysis to achieve a clear classification of SST+ INs. Specifically, morphological reconstructions (Figures 1A,B) were used as the primary criterion for cell-type classification, allowing us to examine axonal arborization patterns and their projections to specific hippocampal layers. Cells morphologically resembling OLM INs possessed elongated axons reaching the SLM (Figure 1A), whereas non-OLM cells displayed shorter axonal projections reaching the SO and stratum radiatum (SR) (Figure 1B). Firing pattern analysis was performed for all recorded cells and included assessment of multiple electrophysiological properties, including RMP, τm, firing frequency, APt, AP amplitude, spike half-width, AHP amplitude, sag potential, and Ri, with particular emphasis on sag potential differences (Table 2). OLM cells displayed a prominent sag potential (18.08 ± 2.07 mV; Figure 1C), whereas non-OLM cells showed a substantially smaller sag potential (3.75 ± 0.58 mV), which was often barely discernible in electrophysiological recordings (Figure 1D). For morphologically ambiguous cells, a sag potential > 9.7 mV (range: 9.7–37.7 mV) was used as an additional criterion supporting their classification as OLM INs, whereas a sag potential < 6.9 mV supported classification as non-OLM cells. Consideration of these morphological and electrophysiological features allowed us to classify these neurons into two SST+ subtypes: OLM and non-OLM INs (Maccaferri and McBain, 1996; Chamberland et al., 2024). Additionally, OLM cells were characterized by a significantly more negative RMP, larger τm, higher APt, and greater AP amplitude than non-OLM cells (Table 2).

**Table 2:** Comparison of mean firing-pattern parameters between OLM and non-OLM INs.

Parameters	OLM cells	non-OLM cells	*p*-value
RMP (mV)	−57.1 ± 1.23	−51.6 ± 1.47	*p* = 0.006**
τm (ms)	31.8 ± 2.75	22.0 ± 2.54	*p* = 0.006**
Firing frequency (Hz)	33.5 ± 2.25	29.1 ± 3.74	*p* = 0.318 ns
APt (mV)	−45.2 ± 1.06	−37.8 ± 3.00	*p* = 0.003**
AP amplitude (mV)	68.6 ± 1.87	58.6 ± 2.53	*p* = 0.003**
spike half-width (ms)	1.2 ± 0.07	1.5 ± 0.08	*p* = 0.056 ns
AHP amplitude (mV)	−23.4 ± 1.13	−15.1 ± 3.60	*p* = 0.239 ns
sag potential (mV)	18.1 ± 2.07	3.7 ± 0.58	*p* < 0.001***
Ri (MΩ)	467.4 ± 42.47	361.2 ± 38.65	*p* = 0.037*

Parameters evaluated were RMP, τ_m_, firing frequency, APt, AP amplitude, spike half-width, AHP amplitude, sag potential, and Ri. *p < 0.05, **p < 0.01, ***p < 0.001; ns, non-significant.

### 3.2 Distinct subtypes of SST+ INs exhibit different tonic current density

Given that tonic GABA_A_R-mediated inhibition has been previously characterized in PCs and various types of INs within the hippocampus (Wyroślak et al., 2021; Wyroślak et al., 2023), revealing marked differences across neuronal subtypes, we first assessed tonic current densities among the two subpopulations of SST+ INs under control conditions. In both OLM (Figure 2A) and non-OLM (Figure 2B) groups, etomidate (3 μM) was applied to enhance the tonic conductance, and tonic current density was quantified as the shift in holding current relative to the zero-current level established following application of the GABA_A_R antagonist PTX (100 μM). The mean raw tonic current was significantly greater in OLM INs (45.73 ± 11.21 pA, *n* = 10, *N* = 9) than in non-OLM cells (18.57 ± 3.02 pA, *n* = 13, *N* = 11, *p* = 0.044; Figures 2A,B). Considering that the values of tonic currents show substantial cell-to-cell variability, primarily due to differences in cell membrane surface, we focused our analysis on tonic current density, calculated as PTX-induced current shift divided by the value of the Cm. We observed that OLM cells exhibited a significantly higher tonic current density compared to non-OLM cells (OLM: 0.70 ± 0.20 pA/pF, *n* = 10, *N* = 9; non-OLM: 0.27 ± 0.04 pA/pF, *n* = 13, *N* = 11, *p* = 0.006; Figure 2C). This difference may indicate a differential expression of extrasynaptic GABA_A_Rs between these SST+ IN subtypes, which could contribute to distinct regulation of cellular and circuit excitability.

**Figure 2:**
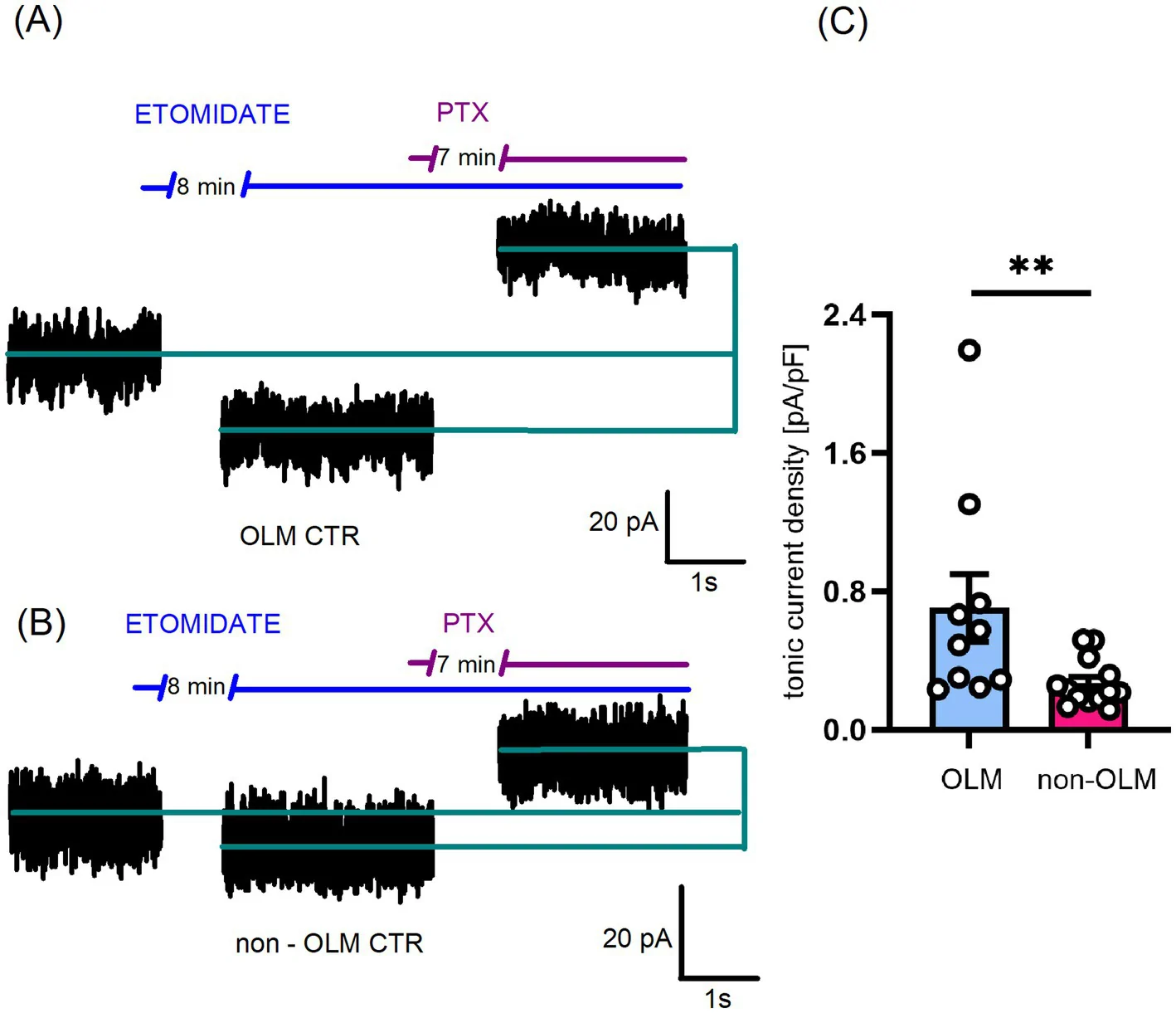
OLM and non-OLM SST+ cell subtypes show different tonic current densities **(A, B)**. Representative tonic current traces recorded from OLM **(A)** and non-OLM INs **(B)** in control conditions. Timing of etomidate and PTX application are indicated by horizontal blue and purple lines, respectively. Dark cyan lines denote the averaged baseline current level before and after compound application. **(C)** Statistics for tonic current density in control conditions for OLM cells (light blue, *n* = 10, *N* = 9) and for non-OLM cells (pink, *n* = 13, *N* = 11). Statistical analysis was performed using the unpaired *t*-test (**p < 0.01). Data on the plots are presented as mean ± SEM, with circles denoting measurements from individual cells. *n*, numbers of cells; *N*, numbers of animals.

### 3.3 Distinct NMDA-induced tonic current plasticity in OLM and non-OLM SST+ INs

Previous studies have shown that a brief, 3-min NMDA application induces long-term synaptic plasticity as well as plasticity of GABAergic tonic currents, in PCs and various types of hippocampal INs, including SST+ INs (Wiera et al., 2021; Brzdąk et al., 2023; Wyroślak et al., 2023). We therefore checked whether NMDA treatment led to differential plastic effects on tonic current in the two SST+ IN subtypes. After achieving stable baseline recordings, NMDA (20 μM) was administered for 3 min to evoke plasticity in OLM and non-OLM cells. Tonic current (enhanced by etomidate) was measured in reference to zero-current level upon PTX treatment at the 18-min time point after the end of NMDA application (Figure 3A). The obtained results did not reveal any significant change in the mean value of tonic current density after plasticity induction in the OLM group (*p* = 0.734; CTR: 0.71 ± 0.2 pA/pF, *n* = 10, *N* = 9; NMDA: 0.65 ± 0.24 pA/pF, *n* = 10, *N* = 10; Figure 3B). Similarly, although non-OLM INs showed a tendency toward higher tonic current density following NMDA treatment, the difference did not reach statistical significance when the entire population was analyzed (*p* = 0.589; CTR: 0.27 ± 0.04 pA/pF, *n* = 13, *N* = 11; NMDA: 0.48 ± 0.11 pA/pF, *n* = 18, *N* = 16; Figure 3D). The absence of significance within the non-OLM population was likely attributable to the substantial variability, suggesting a considerable degree of heterogeneity within this group. To further examine this possibility, cumulative distribution analyses were performed. As shown in Figure 3E, NMDA-treated non-OLM cells exhibited a rightward shift in tonic current density distribution relative to control conditions, primarily in the upper half of the distribution (above the median; hereafter referred to as the upper-median group). This finding raises the possibility that NMDA treatment may preferentially affect non-OLM cells characterized by relatively large tonic currents, despite the lack of a significant effect at the level of the entire group (Supplementary Table S1). Consistent with this interpretation, Mann–Whitney analysis demonstrated a significant increase in tonic current density in the upper-median group (*p* = 0.024; Figure 3F), whereas no difference was observed in the lower-median group (*p* = 0.582; Figure 3G). However, this exploratory stratification should not be interpreted as definitive evidence for the existence of distinct biological subpopulations. Importantly, no significant differences between the upper-median and lower-median groups were detected in the electrophysiological parameters examined, and no distinct axonal arborization patterns were observed. All cells displayed morphological features typical of non-OLM cells, as illustrated in Figure 1B. In contrast, cumulative distribution analysis in the OLM group showed no clear shift following NMDA treatment (Figure 3C). Accordingly, Mann–Whitney test and unpaired *t*-test confirmed the absence of significant differences in either upper-median or lower-median subgroups (*p* = 0.690 and *p* = 0.469, respectively; Figures 3H,I).

**Figure 3:**
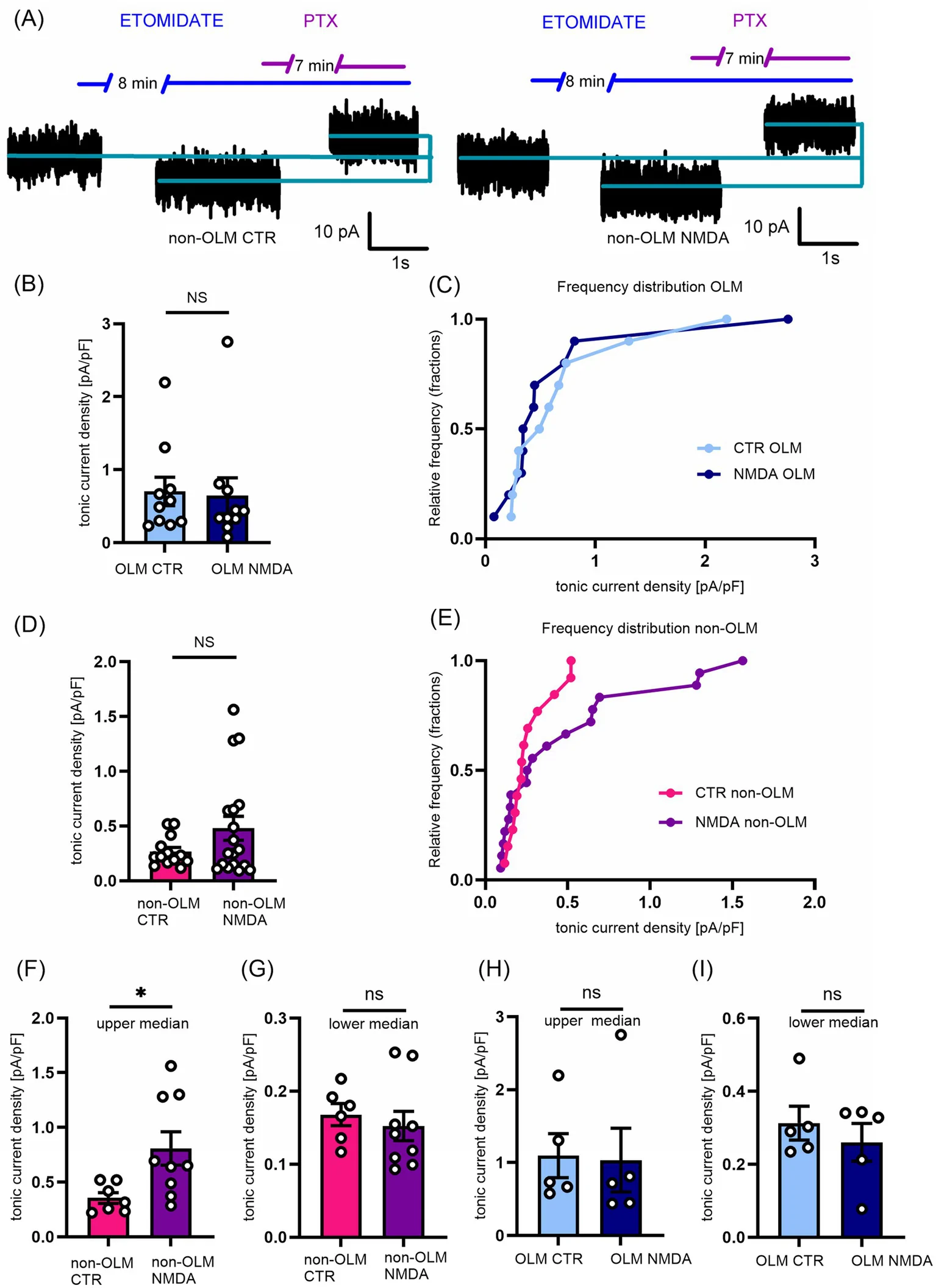
Heterogeneity of NMDA-induced plasticity of tonic GABAergic currents in OLM and non-OLM SST+ INs. **(A)** Representative tonic current traces recorded from non-OLM cells under control conditions (left) and after NMDA treatment (right). Insets above the traces indicate the timing of etomidate (blue) and PTX (purple) applications. The dark cyan lines mark the averaged baseline current level. **(B)** Quantification of tonic current density in OLM INs under control conditions (light blue, *n* = 10; *N* = 9) and after NMDA treatment (dark blue, *n* = 10; *N* = 10). **(C)** Cumulative frequency distribution of the tonic current density in OLM INs under control conditions (light blue line, *n* = 10; *N* = 9) and following NMDA treatment (navy blue line, *n* = 10; *N* = 10). **(D)** Statistical analysis of tonic current density in non-OLM cells, illustrating the effect of NMDA-induced plasticity (purple, *n* = 18; *N* = 16) compared with control conditions (pink, *n* = 13, *N* = 11). **(E)** Cumulative frequency distribution of the tonic current density in non-OLM cells under control conditions (pink line, *n* = 13; *N* = 11) and after NMDA application (purple line, *n* = 18; *N* = 16). **(F)** Summary of tonic current density changes in the upper-median group of non-OLM INs in control group (pink bar, *n* = 7; *N* = 7) and after plasticity induction (purple bar, *n* = 9; *N* = 8). **(G)** Tonic current density measured 16–18 min after NMDA application (purple bar, *n* = 9; *N* = 8), compared with baseline control conditions (pink bar, *n* = 6; *N* = 4), in the lower-median subgroup of non-OLM INs. **(H)** The effect of NMDA administration on tonic current density, 16–18 min after iLTP induction (navy blue bar, *n* = 5; *N* = 5), compared to the control conditions (light blue bar, *n* = 5; *N* = 5), in the upper-median subgroup of OLM cells. **(I)** Summary plots showing the lack of changes in tonic current density in the lower-median subgroup of OLM INs 16–18 min after NMDA treatment (navy blue bar, *n* = 5; *N* = 5) compared to baseline conditions (light blue bar, *n* = 5; *N* = 4). For panels **(F–I)**, cells were divided *post hoc* into upper- and lower-median groups based on the distribution of tonic current density. Analysis was conducted with the Kolmogorov–Smirnov test **(C, E)**, the Mann–Whitney *U* test **(B, D, F, H)** and unpaired *t*-test **(G, I)**.*p < 0.05, ns, non-significant; *n*, numbers of cells; *N*, numbers of animals. Data on the plots **(B, D, F-I)** are presented as mean ± SEM and circles represent values collected in recordings from separate cells.

Taken together, our results demonstrate that non-OLM cells display lower baseline tonic current density than OLM INs, while median-based analysis indicates that the upper-median group of non-OLM cells exhibits a marked susceptibility to NMDA-induced plasticity. In contrast, OLM cells appear resistant to such modulation. These findings highlight the heterogeneity of NMDA-induced plasticity of tonic inhibition among SST+ INs in the hippocampus.

### 3.4 Induction of iLTP increases the relative contribution of α5-GABA_A_Rs to tonic currents in non-OLM SST+ INs

Taking into account the NMDA-induced plastic changes in the tonic current density observed in the non-OLM group (its subpopulation represented by the upper-median) of SST+ INs, but not in OLM cells, we sought to investigate this phenomenon in greater detail. Tonic inhibition in the hippocampus is primarily mediated by δ- and α5-GABA_A_Rs, however, in SST+ INs plasticity of tonic currents appears to arise predominantly from alterations in α5-GABA_A_R content (Wyroślak et al., 2023). We thus tested whether the divergent effects of NMDA treatment on GABAergic tonic currents in OLM and non-OLM INs involved α5-GABA_A_Rs. To this end, we applied L-655,708 (20 nM), a selective inverse agonist at the benzodiazepine binding site of α5-GABA_A_Rs, in both control and iLTP (upper-median) groups to check its effect on etomidate-enhanced tonic currents (Figure 4A). As illustrated in Figure 4B, L-655,708 substantially diminished tonic currents in non-OLM INs under both control and NMDA-treated conditions (CTR: *p* = 0.003; before: 0.40 ± 0.06 pA/pF, after 0.19 ± 0.04 pA/pF, *n* = 5, *N* = 5; NMDA: *p* = 0.014; before: 0.91 ± 0.22 pA/pF, after: 0.58 ± 0.15 pA/pF, *n* = 6, *N* = 6). Conversely, in OLM INs, the α5-GABA_A_R inverse agonist had no effect on tonic current density under control conditions but significantly attenuated tonic currents following NMDA application (CTR: *p* = 0.063; before: 0.63 ± 0.19 pA/pF, after 0.22 ± 0.11 pA/pF, *n* = 5, *N* = 4; NMDA: *p* = 0.024; before: 0.38 ± 0.03 pA/pF, after 0.21 ± 0.06 pA/pF, *n* = 5, *N* = 5; Figure 4C). Despite these effects, L-655,708-sensitive tonic current density did not differ significantly between control and NMDA-treated groups in either non-OLM (*p* = 0.052; CTR: 0.19 ± 0.04 pA/pF, *n* = 5, *N* = 5; NMDA: 0.57 ± 0.15 pA/pF, *n* = 6, *N* = 6; Figure 4D) or OLM INs (*p* = 0.957; CTR: 0.22 ± 0.11 pA/pF, *n* = 5, *N* = 4; NMDA: 0.21 ± 0.06 pA/pF *n* = 5, *N* = 5; Figure 4E). However, a trend toward potentiation after iLTP induction was observed in the non-OLM group. Interestingly, analysis of the mean percentage contribution of the α5-dependent component to total tonic current density revealed a significantly greater contribution of α5-GABA_A_R mediated currents in the iLTP group compared to the control group in non-OLM-classified cells (*p* = 0.039; CTR: 47.47 ± 5.16%, *n* = 5, *N* = 5; NMDA: 62.42 ± 3.70%, *n* = 6, *N* = 6; Figure 4F). In OLM INs, a similar trend was observed but did not reach statistical significance (*p* = 0.396; CTR: 36.77 ± 14.99%, *n* = 5, *N* = 4; NMDA: 54.95 ± 13.68% *n* = 5, *N* = 5; Figure 4G). To further examine the relationship between tonic GABA_A_R-mediated conductance and the contribution of α5-GABA_A_Rs within individual NMDA-treated non-OLM INs, we performed a correlation analysis between tonic current density and the absolute L-655,708-sensitive component. Tonic current density was strongly and positively correlated with the L-655,708-sensitive component (*r* = 0.95, *p* = 0.003, *n* = 6, *N* = 6; Figure 4H), indicating that larger NMDA-induced increases in tonic current density were associated with a larger absolute L-655,708-sensitive component. These data further support the contribution of α5-GABA_A_Rs to tonic GABAergic conductance in non-OLM INs. Together, these findings indicate that transient NMDA administration, a protocol known to induce heterosynaptic GABAergic plasticity, was also found to enhance tonic currents in non-OLM SST+ cells, accompanied by an increased relative contribution of α5-GABA_A_Rs (Wyroślak et al., 2021, 2023).

**Figure 4:**
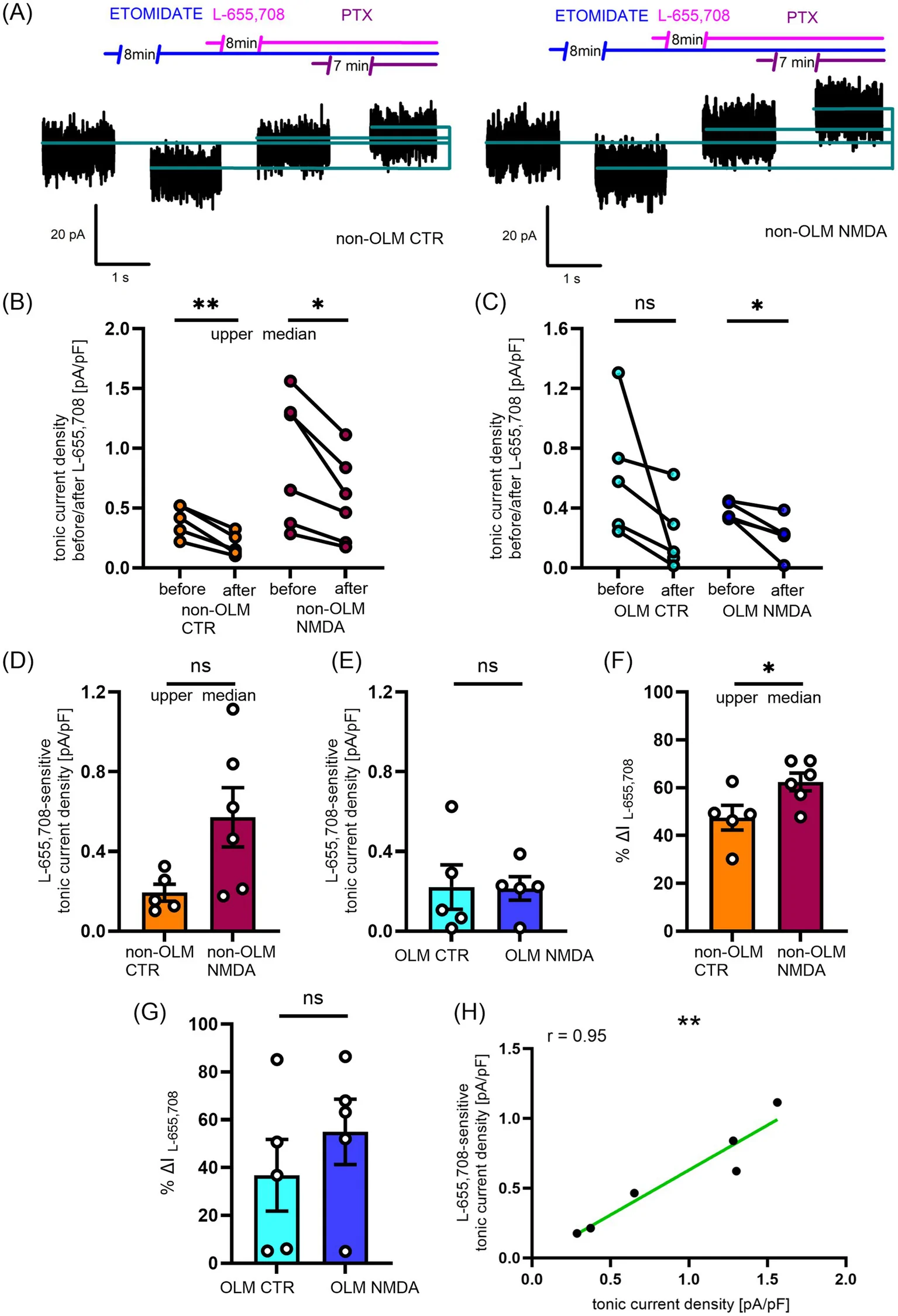
Induction of long-term inhibitory plasticity increases the mean percentage of L-655,708-sensitive tonic current mediated by α5-GABA_A_Rs in non-OLM INs. **(A)** Representative current traces from the control (left) and iLTP groups (right) in non-OLM INs. **(B, C)** Tonic current density in individual cells before and after L-655,708 application in the upper-median group of non-OLM cells under control conditions (orange, *n* = 5; *N* = 5) and after NMDA application (dark pink, *n* = 6; *N* = 6) **(B)**, and in OLM cells under control conditions (cyan, *n* = 5; *N* = 4) and after NMDA administration (dark blue, *n* = 5; *N* = 5) **(C)**. Tonic current density was calculated as the difference between the holding current measured after etomidate application and after PTX application, normalized to whole-cell membrane capacitance (Cm). The tonic current density before L-655,708 application represents the total tonic GABA current density. **(D)** L-655,708-sensitive tonic current density in upper-median group of non-OLM INs in control conditions (orange, *n* = 5; *N* = 5) and after NMDA administration (dark pink, *n* = 6; *N* = 6). **(E)** L-655,708-sensitive tonic current density in OLM cells in control group (cyan, *n* = 5; *N* = 4) and after plasticity induction (dark blue, *n* = 5; *N* = 5). **(F)** Percentage contribution of the α5-dependent component to the tonic current density in the upper-median group of non-OLM cells in control group (orange, *n* = 5; *N* = 5) and after NMDA treatment (dark pink, *n* = 6; *N* = 6). **(G)** Statistics of the mean percentage contribution of the α5-dependent component to the tonic current density in OLM cells in control group (cyan, *n* = 5; *N* = 4) and after NMDA treatment (dark blue, *n* = 5; *N* = 5). **(H)** Correlation between tonic current density and the L-655,708-sensitive component in non-OLM SST+ INs from the upper-median group (black dots, *n* = 6; *N* = 6). The green line represents the linear trend.*p < 0.05, **p < 0.01; ns, non-significant, *n*, numbers of cells; *N*, numbers of animals.

## 4 Discussion

The major finding of the present study is that distinct subclasses of SST+ INs show differences in tonic current density and its susceptibility to plastic changes induced by exogenous NMDA. Specifically, using combined electrophysiological characterization and morphological reconstruction, we classified SST+ INs into OLM and non-OLM subgroups (Figure 1) and found that tonic current density in the former subclass is markedly larger than in the latter one (Figure 2), further underscoring their functional heterogeneity.

Our data extend previous observations demonstrating that tonic inhibition exhibits heterosynaptic, NMDAR-dependent, and cell type-specific plasticity in PCs and various classes of hippocampal INs (Wyroślak et al., 2021, 2023). In particular, we have previously shown that in hippocampal CA1 SST+ INs, enhancement of tonic conductance is associated with an increased contribution of α5-GABA_A_Rs (Wyroślak et al., 2023). In the present study we have found that this plasticity is not uniform across all SST+ INs. Whereas tonic current density remained unchanged in OLM cells (Figures 3C,H,I), transient NMDA application was associated with an increase in tonic current density in the upper-median group of non-OLM cells identified by an exploratory, *post hoc* median-based stratification (Figures 3E,F).

Importantly, the NMDA-induced alterations in tonic current density observed in a subset of non-OLM cells were associated with a significant increase in the percentage contribution of α5-GABA_A_Rs to total tonic current density (Figure 4F), consistent with our previous observations for the entire SST+ IN population (Wyroślak et al., 2023). The finding that NMDA-induced enhancement of tonic inhibition was restricted to a subset of non-OLM INs further emphasizes the heterogeneity of this population and suggests that the morphological and electrophysiological criteria used in the present study are insufficient to resolve all functionally distinct SST+ IN subtypes. In contrast, OLM cells did not exhibit any statistically significant alterations or even any discernible trend toward tonic current changes following NMDA application (Figures 3B,C,H,I), indicating that tonic inhibitory signaling in these SST+ IN subtypes is resistant to this form of plasticity. Interestingly, correlation analysis revealed a strong positive relationship between tonic current density and the L-655,708-sensitive component in non-OLM INs (*r* = 0.95, *p* = 0.003). This finding indicates that cells with larger tonic currents also exhibited a greater α5-GABA_A_R-mediated component, supporting a contribution of α5-GABA_A_Rs to tonic GABAergic conductance in non-OLM INs. Our findings show some quantitative difference with respect to our previous investigation (Wyroślak et al., 2023), in which we observed a significant enhancement of tonic current density following plasticity induction for the SST+ INs analyzed as a single population. A likely explanation for this discrepancy is the pronounced heterogeneity of SST+ INs, as averaging across multiple functionally distinct subtypes may obscure subtype-specific forms of plasticity. In addition, to minimize the non-specific effects of etomidate (used to enhance tonic currents), we decided to reduce its concentration from 5 μM (Wyroślak et al., 2023) to 3 μM in the present study. Although the lower concentration likely improved specificity of this compound, it may also have reduced the detectability of smaller plasticity-related changes. Taken altogether, these findings demonstrate that extrasynaptic inhibitory plasticity is highly cell-type-specific within SST+ IN populations.

The distinct morphological characteristics and tonic inhibitory properties observed in SST+ IN subpopulations are consistent with the well-established diversity of these cells in terms of their electrophysiological properties, morphology, connectivity, and functional roles within hippocampal circuits (Pelkey et al., 2017; Booker and Vida, 2019; Chamberland et al., 2024). Among SST+ INs, OLM cells represent a particularly important subtype that selectively targets distal dendrites of CA1 pyramidal neurons and regulates information flow from the entorhinal cortex (Udakis et al., 2025). In contrast, other SST+ IN populations primarily participate in local (except for SST+ projection cells) inhibitory and disinhibitory microcircuits within the hippocampus (Honoré et al., 2021; Takács et al., 2024). Importantly, the non-OLM group includes several functionally distinct subclasses. For example, Sst: Tac1 INs preferentially inhibit fast-spiking INs, whereas Sst: Nos1 cells appear unable to interrupt fast-spiking INs’ activity (Chamberland et al., 2024). Such functional diversity likely contributes to differences in both basal tonic inhibition and the mechanisms governing its plasticity. In this context, it is particularly important to note that NMDA receptor signaling can engage multiple intracellular pathways capable of differentially modulating tonic GABAergic inhibition depending on cellular context and receptor composition (Wu et al., 2021). It is also noteworthy that OLM INs appear to possess a distinct NMDA receptor profile compared with other SST+ cells in the hippocampus. In particular, GluN2A-containing NMDA receptors play a prominent functional role in OLM cells, distinguishing them from other SST+ IN subtypes (Booker et al., 2021). Consequently, analyses that do not distinguish among SST+ IN subtypes may oversimplify the underlying mechanisms and potentially obscure functionally relevant forms of cell type-specific plasticity. Consistent with this notion, our observations indicate that non-OLM SST+ INs are characterized by relatively low basal tonic current density but exhibit a measurable susceptibility to NMDA receptor-dependent tonic inhibitory plasticity. In contrast, OLM INs appear to display a larger tonic current density but are less prone to regulation by NMDA, further emphasizing the marked functional heterogeneity within the SST+ IN populations. Such differences in basal values of tonic current density and its plasticity may contribute to cell type-specific regulation within hippocampal SST+ IN populations. Interestingly, an analogous cell type-dependent modulation by tonic conductance has been found, for instance, in the barrel cortex where it has been demonstrated that tonic GABAergic signaling in SST+ INs exerts a less pronounced effect on intrinsic excitability than in INs expressing vasoactive intestinal polypeptide (VIP) (Bogaj et al., 2023). We may speculate that the distinct connectivity profiles of OLM and non-OLM INs and their different features of tonic inhibition may support distinct network functions, with OLM-mediated tonic conductance potentially tuned to regulate distal entorhinal input, whereas other SST+ populations contribute primarily to local inhibitory processing (Takács et al., 2024).

A particularly interesting finding of the present study is that NMDA-dependent plasticity of tonic current density in non-OLM INs is associated with an altered percentage of α5-GABA_A_Rs. This GABA_A_R subtype was found to be particularly abundant in the hippocampus, especially in CA1 PCs, where it strongly influences neuronal excitability and synaptic integration (Fritschy and Mohler, 1995; Collinson et al., 2002; Caraiscos et al., 2004). Accumulating evidence further suggests that α5-GABA_A_Rs are involved in learning and memory processes (Collinson et al., 2006; Martin et al., 2010; review: Nagarajan et al., 2026) and could be a drug target for cognitive enhancement (Collinson et al., 2002; Braudeau et al., 2011; Donegan et al., 2019; O'Connell and Kwakowsky, 2026). The functional specialization of α5-GABA_A_Rs is supported by their subcellular organization. Unlike more diffusely distributed receptor populations, α5-GABA_A_Rs form extrasynaptic clusters through interactions with gephyrin- and radixin-associated scaffolding complexes (Jacob, 2019), a feature that likely contributes to cell type-specific regulation of tonic inhibitory signaling. Moreover, the relocation of these receptors between extrasynaptic and synaptic localizations has been proposed as a key mechanism underlying inhibitory plasticity (Hausrat et al., 2015; Kneussel and Hausrat, 2016; Davenport et al., 2021). This mechanism was initially characterized in hippocampal pyramidal neurons (e.g., Hausrat et al., 2015; Kneussel and Hausrat, 2016; Davenport et al., 2021), but other studies indicated its presence also in hippocampal GABAergic INs including SST+ subclass (Wiera et al., 2021; Brzdąk et al., 2023; Wyroślak et al., 2023). In addition, the presence of α5-GABA_A_Rs in CA1 SST+ INs has previously been reported (Salesse et al., 2011; Magnin et al., 2019), as well as in other IN subclasses (Petrache et al., 2020). However, tonic currents in dentate gyrus granule cells are primarily mediated by δ-subunit-containing receptors (Glykys et al., 2008) and in the hippocampal PV+ fast-spiking INs the contribution of these receptors is also substantial (Wyroślak et al., 2023), showing that distinct GABA_A_R subtypes may contribute differentially to tonic inhibition depending on neuronal identity. Importantly, α5-GABA_A_Rs appear to serve distinct functions depending on their cellular localization. It has been demonstrated that phasic inhibition mediated by VIP/CR IN inputs onto SST+ INs regulates anxiety-like behavior, whereas tonic inhibition mediated by SST+ INs onto PCs contributes to spatial learning (Magnin et al., 2019). Consistent with these observations, several studies supported a critical role of tonic inhibition and extrasynaptic α5-GABA_A_Rs in hippocampus-dependent learning, memory formation, and inhibitory synaptic plasticity (Engin et al., 2015; Möhler and Rudolph, 2017; Donegan et al., 2019; Davenport et al., 2021; O'Connell et al., 2026). Recent evidence further suggested that α5-GABA_A_Rs, particularly those expressed by INs, play a critical role in regulating hippocampal network dynamics and contextual memory processing through tonic inhibitory mechanisms (Zhu et al., 2023). Taken together, differences in the relative contribution of the L-655,708-sensitive component to tonic current across SST+ IN populations may contribute to differences in the regulation of tonic inhibition and, consequently, to distinct aspects of neuronal network function. It is worth noting that, in the present study, the L-655,708-sensitive component is consistent with a contribution of α5-GABA_A_Rs to tonic inhibition in these cells. Importantly, however, the present experiments do not provide direct evidence for changes in α5-GABA_A_R abundance or expression. The observed increase in the relative contribution of the L-655,708-sensitive component may reflect changes in receptor function, receptor localization or distribution, or alterations in other components contributing to tonic GABA_A_R-mediated currents. These possibilities should therefore be considered as potential mechanisms rather than conclusions directly supported by the present experiments. An important consideration when interpreting the present findings is the developmental age of the animals used for electrophysiological recordings (P20–P24). Although this age is beyond the period of the most pronounced early postnatal maturation of SST+ INs and many intrinsic electrophysiological properties are already well established by this stage (Pan et al., 2016), P20–P24 mice cannot be considered fully adult but rather as juvenile adult, as some features of GABAergic transmission in rodents may continue to undergo modifications beyond this time window (Banks et al., 2002; Ben-Ari et al., 2007). The use of this age range was also consistent with our previous study of tonic GABAergic inhibition in hippocampal SST+ INs (Wyroślak et al., 2023), allowing methodological continuity and comparison between studies. Future studies using adult animals will be required to determine whether the mechanisms identified here are fully preserved following further developmental maturation. Another methodological consideration concerns the use of a non-physiological, high-KCl internal solution during current-clamp recordings. The elevated intracellular Cl^−^ concentration may affect the time course and threshold of action potentials (e.g., Sørensen et al., 2017) and therefore should be considered when interpreting the electrophysiological properties of INs measured in current-clamp. Although action potentials are primarily determined by voltage-gated Na^+^ and K^+^ conductances, potential contributions of Cl^−^ currents to firing properties cannot be excluded under our recording conditions.

Overall, our results demonstrate substantial heterogeneity in tonic inhibition and its plasticity across CA1 SST+ INs. To our knowledge, this study provides the first evidence that OLM and non-OLM SST+ INs differ substantially in both the basal properties and plasticity mechanisms of tonic inhibition. These findings highlight the importance of incorporating SST+ INs heterogeneity into models of inhibitory circuit function, as subtype-specific functions and modulation of tonic inhibition may critically influence dendritic excitability, synaptic integration, and hippocampal network computations related to learning and memory. In particular, our results support growing evidence from single-cell transcriptomic, functional and morphological studies indicating extensive neuronal diversity underlying circuit-level computations (Gouwens et al., 2020; Yao et al., 2023; Brzdąk et al., 2025). Importantly, these observations may also have translational relevance as α5-GABA_A_Rs, shown here to be involved in tonic current plasticity, are considered promising therapeutic targets for neurological and psychiatric disorders (Nuwer et al., 2023; O'Connell et al., 2026; Troppoli and Thompson, 2026; Uliana et al., 2026).

## Data Availability

The datasets analyzed in this study are publicly available in the Polska Platforma Medyczna Uniwersytetu Medycznego we Wrocławiu (PPM-UMW) repository, DOI: https://doi.org/10.60956/fcah-x309, and all other raw data are available from the corresponding author upon reasonable request.

## Abbreviations

GABA_A_Rs: GAB_A_A receptors
INs: interneurons
mIPSC: miniature inhibitory postsynaptic current
OLM: oriens-lacunosum moleculare
SST+: somatostatin-positive
α5-GABA_A_Rs: α5 subunit-containing GABA_A_Rs
GABA: γ-aminobutyric acid
iLTP: inhibitory long-term potentiation
PV+: parvalbumin-positive
PCs: pyramidal cells
NMDA: N-methyl-D-aspartic acid
SO: stratum oriens
SP: stratum pyramidale
SR: stratum radiatum
SLM: stratum lacunosum-moleculare
ACSF: artificial cerebrospinal fluid
TTX: tetrodotoxin
DNQX: 6,7-dinitroquinoxaline-2,3-dione
PTX: picrotoxin
PBS: phosphate-buffered saline
NHS: normal horse serum
RMP: resting membrane potential
τm: membrane time constant
APt: action potential threshold
AP amplitude: action potential amplitude
AHP: afterhyperpolarization
Ri: input resistance
Cm: membrane capacitance
VIP: vasoactive intestinal polypeptide
