# Supplementary material for: Distinct tonic inhibition and its plasticity in hippocampal OLM and non-OLM somatostatin-positive interneurons
Source: Front Mol Neurosci. 2026 Oct 30;19:1934043. doi: 10.3389/fnmol.2026.1934043 (PMC13759832; doi:10.3389/fnmol.2026.1934043)
Supplement: Supplementary file 1 [file Data_Sheet_1.DOCX]

Supplementary Material

Figure. S1. Representative current traces from control (S1A) and iLTP (S1B) non-OLM interneurons (INs). Insets above the traces indicate the timing of etomidate (blue), L-655,708 (pink), and PTX (purple) applications. Dark cyan lines indicate the averaged baseline current level.

|  | **non-OLM CTR** | **non-OLM NMDA** |
| --- | --- | --- |
| **Total number of values** | 13 | 18 |
| **Number of excluded values** | 0 | 0 |
| **Number of binned values** | 13 | 18 |
|  |  |  |
| **Minimum** | 0,117 | 0,093 |
| **25% Percentile** | 0,173 | 0,136 |
| **Median** | 0,220 | 0,270 |
| **75% Percentile** | 0,368 | 0,662 |
| **Maximum** | 0,520 | 1,563 |
|  |  |  |
| **Mean** | 0.269 | 0.481 |
| **Std. Deviation** | 0.136 | 0.462 |
| **Std. Error of Mean** | 0.038 | 0.110 |
|  |  |  |
| **Lower 95% CI of mean** | 0.186 | 0.251 |
| **Upper 95% CI of mean** | 0.351 | 0.710 |
|  | **OLM CTR** | **OLM NMDA** |
| **Total number of values** | 10 | 10 |
| **Number of excluded values** | 0 | 0 |
| **Number of binned values** | 10 | 10 |
|  |  |  |
| **Minimum** | 0.235 | 0.078 |
| **25% Percentile** | 0.279 | 0.299 |
| **Median** | 0.534 | 0.391 |
| **75% Percentile** | 0.877 | 0.742 |
| **Maximum** | 2.195 | 2.755 |
|  |  |  |
| **Mean** | 0.705 | 0.647 |
| **Std. Deviation** | 0.616 | 0.772 |
| **Std. Error of Mean** | 0.195 | 0.244 |
|  |  |  |
| **Lower 95% CI of mean** | 0.264 | 0.095 |
| **Upper 95% CI of mean** | 1.145 | 1.199 |

Table S1. Descriptive statistics of non-OLM and OLM cells.
